# The Regulatory Role of MicroRNAs in Breast Cancer

**DOI:** 10.3390/ijms20194940

**Published:** 2019-10-06

**Authors:** Hui-Yi Loh, Brendan P. Norman, Kok-Song Lai, Nik Mohd Afizan Nik Abd. Rahman, Noorjahan Banu Mohamed Alitheen, Mohd Azuraidi Osman

**Affiliations:** 1 Department of Cell and Molecular Biology, Faculty of Biotechnology and Biomolecular Sciences, Universiti Putra Malaysia, Serdang, Selangor 43400, Malaysia; lohhuiyi.94@gmail.com (H.-Y.L.); m.afizan@upm.edu.my (N.M.A.N.A.R.); noorjahan@upm.edu.my (N.B.M.A.); 2 Department of Musculoskeletal Biology, Institute of Ageing and Chronic Disease, University of Liverpool, Liverpool L7 8TX, UK; Brendan.Norman@liverpool.ac.uk; 3 Health Sciences Division, Abu Dhabi Women’s College, Higher Colleges of Technology, Abu Dhabi 41012, UAE; laikoksong@gmail.com

**Keywords:** microRNAs, breast cancer, oncomiR, tsmiR

## Abstract

MicroRNAs (miRNAs) are small non-coding RNA molecules which function as critical post-transcriptional gene regulators of various biological functions. Generally, miRNAs negatively regulate gene expression by binding to their selective messenger RNAs (mRNAs), thereby leading to either mRNA degradation or translational repression, depending on the degree of complementarity with target mRNA sequences. Aberrant expression of these miRNAs has been linked etiologically with various human diseases including breast cancer. Different cellular pathways of breast cancer development such as cell proliferation, apoptotic response, metastasis, cancer recurrence and chemoresistance are regulated by either the oncogenic miRNA (oncomiR) or tumor suppressor miRNA (tsmiR). In this review, we highlight the current state of research into miRNA involved in breast cancer, with particular attention to articles published between the years 2000 to 2019, using detailed searches of the databases PubMed, Google Scholar, and Scopus. The post-transcriptional gene regulatory roles of various dysregulated miRNAs in breast cancer and their potential as therapeutic targets are also discussed.

## 1. Introduction

MicroRNAs are a family of evolutionarily conserved small, endogenous, single-stranded and non-protein coding RNAs spanning 19 to 25 nucleotides in length [1]. The first miRNA, *lin-4*, was discovered in 1993 as a small RNA transcribed from the lin-4 locus of *Caenorhabditis elegans* [2]. According to the miRBase miRNA sequence database (release 22.0, March 2018) (http://www.mirbase.org/), the human genome contains 2654 mature miRNA sequences to date [3]. miRNAs function as the key post-transcriptional regulators of gene expression in different tissues and developmental stages via highly specific interactions and complex regulatory networks [4].

**Figure 1:**
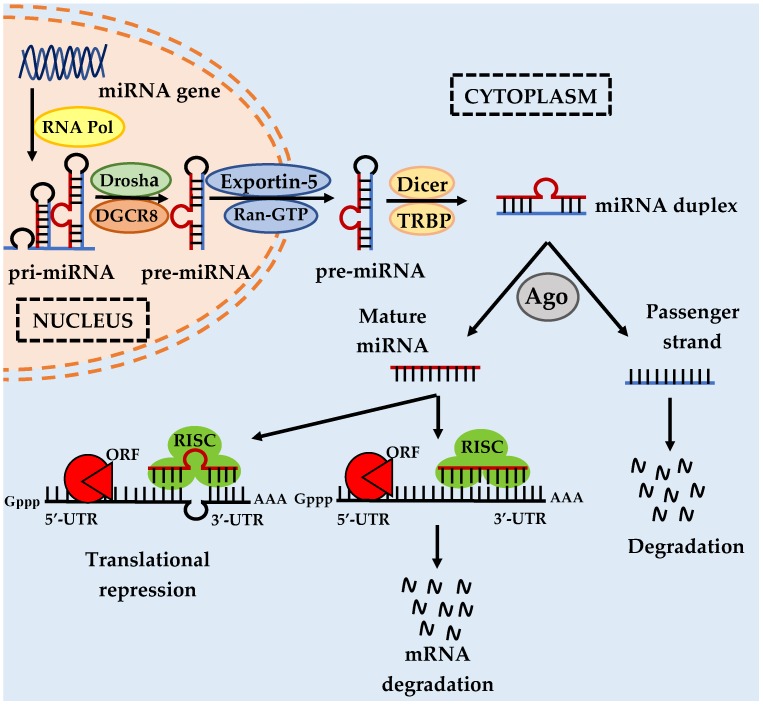
MicroRNA biogenesis and modulation of miRNA activity. miRNA genes are transcribed to produce primary miRNA transcripts (pri-miRNA) by RNA polymerase II. Drosha–DGCR8 complex cleaves the pri-miRNA into a precursor miRNA transcript (pre-miRNA) which is then transported from the nucleus into the cytoplasm via nuclear pore by exportin 5. In the cytoplasm, the pre-miRNA is further modified by the DICER and TRBP complex to form a mature miRNA duplex. The miRNA duplex is incorporated into an Argonaute (Ago) with RNA-induced silencing complex (RISC) and the duplex is unwound by helicase into two single-stranded miRNAs. The mature single-stranded miRNA can then bind to the target mRNA and exert its inhibitory function through translational block or degradation of the mRNA depending on the level of nucleotide complementarity. Reproduced with permission from Bhardwaj, A.; Singh, S.; Singh, A.P. MicroRNA-based cancer therapeutics: Big hope from small RNAs. *Mol. Cell Pharmacol.* 2010 [26].

The mechanisms of miRNA production or biogenesis involve several crucial biological steps starting from miRNA transcription in the nucleus and with further processing and maturation in the cytoplasm. miRNA genes can be intergenic or intragenic. Intergenic miRNA genes are independent, with their own transcription units including promoters, transcript sequences. and terminator units [5,6]. However, intragenic genes are located either in the intronic or exonic regions of host genes, sharing the same transcriptional units with these host genes [6,7]. Intronic miRNAs are found in the introns of non-coding RNA or protein-coding genes, while the exonic miRNAs commonly overlap an exon and an intron of a gene [8,9]. Mirtons are formed when the sequence of the introns of the host genes are identical to the precursor miRNA (pre-miRNA), with splice sites at either end [8,9]. Hence, Drosha microprocessor processing is not essential for maturation of mirtons [10]. Drosha processing is the process of generation of pre-miRNA from primary miRNA (pri-miRNA) in the first step of miRNA biogenesis (Figure 1).

In mammals, miRNA genes are transcribed by RNA polymerase II/III to generate the primary transcripts (pri-miRNAs). Pri-miRNAs typically comprise several thousand nucleotides in length with local stem loop structures, a 5′-cap, and a poly-A tail [11,12]. RNA polymerase II is the major polymerase type for transcription of miRNAs, though there are small groups of miRNAs associated with Alu elements that are transcribed by RNA polymerase III [12,13]. As shown in Figure 1, pri-miRNAs are then processed by a microprocessor complex, Drosha–DiGeorge syndrome critical region gene 8 (DGCR8), into the precursor transcripts (pre-miRNAs), which are approximately 70 nucleotides long and in hairpin form [14,15]. Drosha is a RNase III-type endonuclease that cleaves the pri-miRNA, while DGCR8 is a double-stranded RNA binding protein that acts as a molecular anchor recognizing the pri-miRNA and ensuring correct splicing by Drosha [15].

Pre-miRNAs are then transported from the nucleus into the cytoplasm by RanGTP-dependent nuclear transport reporter exportin 5 (XPO5) to undergo loop-cleavage by another RNase III enzyme known as Dicer, with the aid of transactivation response RNA binding protein (TRBP) for generating an approximately 20 nt-long mature miRNA/miRNA* duplex, as shown in Figure 1 [16,17,18,19]. The miRNA duplexes are then incorporated into a member of the Argonaute (Ago) protein subfamily, facilitated by the Dicer–TRBP complex and resulting in the formation of RNA-induced silencing complex (RISC) [18,19]. The miRNA duplexes are separated or unwound into two single strands by RNA helicases [20]. The guide strand (miRNA mature strand) remains bound to RISC, whereas the passenger strand (miRNA*) undergoes degradation [18]. The Ago protein-bound mature miRNA is subsequently assembled into an effector complex known as the miRNA-containing RNA-induced silencing complex (miRISC) [18]. Within the miRISC, the mature miRNA then binds, with its ‘seed sequence’ (nucleotide 2 to 8 from miRNA 5′-end), to the 3′-UTR (and, in some cases, 5′-UTR and open reading frame (ORF)) of the target messenger RNA (mRNA) [21].

The degree of complementarity between the miRNAs and their mRNA targets determines the inhibitory mechanism of protein expression. Perfect complementary between miRNAs and their mRNA targets induces the degradation of the mRNA [22]. However, partial complementary base pairing between miRNAs and their mRNA targets is more commonly observed, resulting in protein translational repression or inhibition [22]. Due to their short miRNA–mRNA binding site, a single miRNA can bind to multiple targeted mRNAs and regulate their functions in multiple pathways [23,24]. At the same time, a single mRNA can be cooperatively targeted and bound by several different miRNAs [23,24]. It is estimated that approximately one-third of protein-coding genes could be regulated by miRNAs [25]. Hence, the identification of validated targets of miRNAs is of great importance.

Owing to their diverse activity, miRNAs are able to regulate myriad cellular and signaling pathways, including cell development and differentiation, cell proliferation, and apoptosis [27]. Dysregulation of a single miRNA or a small subset of miRNAs can therefore have significant consequences in terms of cellular outcomes and, sometimes, the development of disease processes, including cardiovascular diseases [28], neurodevelopmental diseases [29], autoimmune disorders [30], bone diseases [31], and human cancers such as breast cancer [32,33]. Breast cancer is a complex disease which poses a great challenge to human health, reduces life quality, and causes substantial financial burden across the globe. According to the Global Cancer Project 2018 (GLOBOCAN 2018), female breast cancer ranked as the second most commonly diagnosed cancer and the fifth leading cause of cancer-related death worldwide. It was estimated that 2,088,849 newly identified breast cancer cases (11.6% of 18.1 million new cases) and 626,679 breast cancer-related deaths (6.6% of 9.6 million deaths) occurred globally in the year 2018, including both males and females [34]. Therefore, the prevalence and lethality of breast cancer highlight the importance of investigating the mechanisms involved in breast tumorigenesis, as well as the development of new methods for its prognosis and the identification of new therapeutic targets.

Breast cancer occurs due to abnormal proliferation of any cells or tissues lining the mammary glands and ducts. Most of the breast malignant lesions are carcinomas which can be specifically classified as adenocarcinomas [35]. Breast cancer is a highly heterogeneous disease with diverse intertumoral and intratumoral non-uniformity, and with wide variation in tumors between affected individuals [36]. Currently, breast cancer can also be classified into six molecular intrinsic subtypes: luminal A, luminal B, HER^+^, normal-like, basal (also known as triple-negative), and claudin-low, each based on their unique phenotype, tumor grade, and molecular characterizations including hormone receptors and human EGF-like receptor 2 (HER2) receptor status [37]. Breast cancer is a complex neoplastic disease, comprising the processes of tumor initiation and growth [38], metastasis and invasion [39], and angiogenesis [40], with an additional significant possibility of relapse [41]. These malignant changes occur when the cellular and molecular signaling pathways of the mammary cells are disturbed or dysregulated [42].

The five-year survival rate of breast cancer at stage I, II, III, and IV are 100%, 93%, 72%, and 26% respectively [43,44]. Although earlier diagnosis and detection of breast cancer has led to a decrease in death rates, further advances in prevention, detection, and treatment are urgently required for the improvement of breast cancer outcomes and survival [45]. Conventional breast cancer treatments, such as surgery, radiation, chemotherapy, and hormone therapy, inevitably have side effects regarding their post-treatment reaction or sensation, toxicities, and drug resistance, despite their undeniable effectiveness in the treatment of early-stage breast cancer [46,47]. In recent years, miRNAs have started to attract considerable interest for their regulatory involvement in the initiation, progression, and metastasis of breast cancer [48]. Furthermore, the expression level of certain miRNAs is closely linked to the morphological features, immunohistochemical profiles, histopathological parameters, clinical outcomes, and prognosis and treatment responses of breast cancer [49]. In addition, studies also revealed the presence of aberrant miRNA expression profiles in breast cancer conditions when compared to their non-malignant counterparts [50]. As one of the largest classes of gene regulators, miRNA molecules have vast potential as new biological therapeutic agents, targets, or biomarkers for patient-tailored breast cancer treatment. This review considers the role of miRNAs associated with breast cancer, explores their contributions to the etiology of the disease and discusses the prospects of miRNA-based breast cancer therapeutic strategies.

## 2. Breast Cancer-Linked MicroRNAs

About 50% of the human miRNA-encoding genes are located in the cancer-linked regions or fragile chromosomal sites [24]. Since the role of miRNA dysregulation in breast cancer was first reported in 2005 [51], numerous studies have shown altered expression of miRNAs in breast cancer. These breast cancer-associated miRNAs can be subdivided into the oncogenic miRNAs (oncomiRs) and tumor suppressor miRNAs (tsmiRs), as discussed in the upcoming sections and summarized in Table 1.

**Figure 2:**
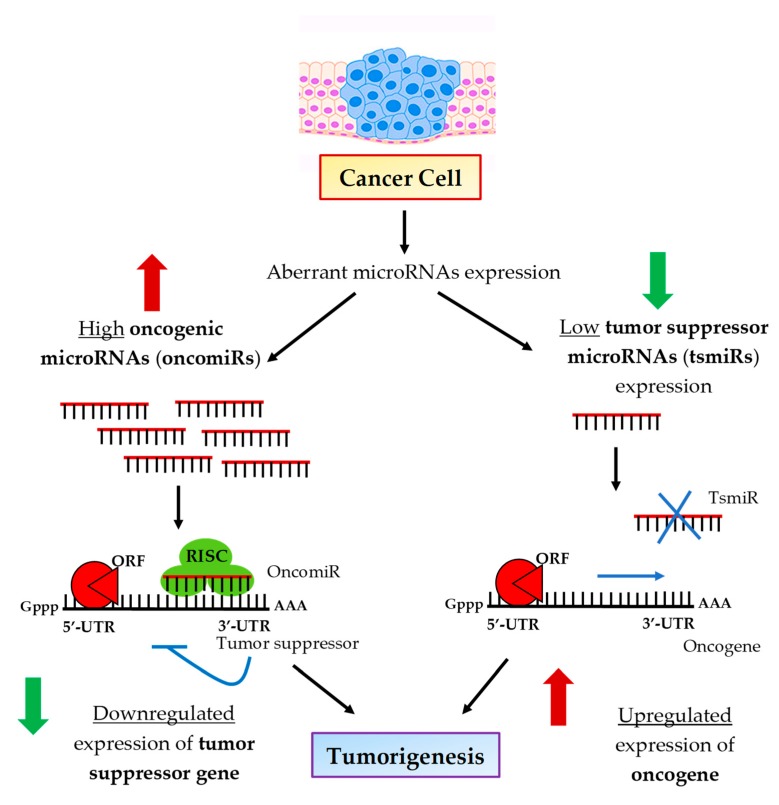
Regulatory mechanisms of oncogenic and tumor suppressor microRNAs in tumorigenic events. Increased expression of oncogenic miRNAs in cancerous cells inhibits tumor suppressor genes. Decreased expression of tumor suppressor miRNAs potentially enhances the expression of oncogenes. Consequently, both oncogenic and tumor suppressor miRNAs lead to tumor development by stimulating cell proliferation, anti-apoptotic response, replicative immortality, invasion, metastasis and angiogenesis. Reproduced with permission from Joshi, M.; Singh Sodhi, K.; Pandey, R.; Singh, J.; Goyal, S.; Dahal, A. MicroRNA: Biomarker for cancer diagnosis and prognosis. *J. Pharm. Biomed. Sci.* 2014 [55].

OncomiRs are usually upregulated in breast cancer, suppressing the expression of potential tumor suppressor genes and leading to breast malignancy [52]. Conversely, tsmiRs can inhibit the expression of oncogenes that promote breast tumorigenesis [53]. Therefore, their downregulation can lead to breast malignancy [52]. Figure 2 illustrates the specific regulatory action of oncomiRs and tsmiRs in tumorigenic events.

Both oncomiRs and tsmiRs critically regulate breast tumor development and progression by participating in complex regulatory networks [52]. These networks include several hallmarks of cancer, such as sustaining growth and proliferative signals, replicative immortality, initiating metastasis and invasion, resisting apoptotic and cell death responses, inducing angiogenesis, activating metabolism or cellular energetics, and supporting cell immune escape [42,54].

## 3. MicroRNAs and the Hallmarks of Breast Cancer

### 3.1. Cell Proliferation and Cell Cycle Regulation

Cell proliferation is the most important hallmark of breast cancer and its dysregulation is the prime cause of breast tumorigenesis [42,54]. Cell cycle progression is essential in maintaining a delicate balance between promoting cell proliferation and its suppression [56]. Healthy cells have a limited capacity for cell division; they have a finite number of cell divisions that is in large determined by reaching an optimum cell density within a tissue. Once a finite cell density is reached, healthy cells will desist from proliferation, arrest at the G0 phase of the cell cycle, and remain quiescent. This behavior of non-cancerous cells is due to the response to the growth inhibitory influences from the environment [57]. This physiologically adaptive cell cycle arrest mechanism is aberrant in cancer cells [58]. Studies have demonstrated that miRNAs have a regulatory role in the multiple cell proliferation and cell cycle progression pathways of breast cancer, by functional interaction with factors such as the cyclin protein family, protein kinases and their inhibitors, and other growth promoters or suppressors.

The *cyclin E1* gene has emerged as an important target for miRNAs that have decreased expression in breast cancer, namely, the tsmiRs miR-497 [59], miR-16 [60], and miR-30c-2-3p [61]. Overexpression of these tumor-suppressive miRNAs was able to inhibit breast cancer cell proliferation and cell cycle progression. Cyclin E1 is an important cell cycle regulator of the G1–S transition [59,60,61]. For instance, Huang and Lyu reported the downregulation of tumor suppressor miR-483-3p in breast cancer cells and its overexpression significantly reduced cell proliferation and inhibited breast cancer G1–S cell cycle transition. *Cyclin E1* was shown to be a direct target of miR-483-3p. Decreased cyclin E1 expression by miR-483-3p overexpression further prevents DNA synthesis initiation by p-NPAT, the downstream target of cyclin E1, which blocks the breast cancer cells from entering the S-phase of the cell cycle. Additionally, the formation of a complex between cyclin E1 and cyclin-dependent kinase CDK2, responsible for cell cycle regulation, was also impeded upon miR-483-3p overexpression [62].

Aside from the increased expression of cyclins, the upregulation of protein kinases and downregulation of its kinase inhibitors by miRNAs can also increase breast cancer cell viability and result in aberration of the cell cycle transition. Zhou et al. reported that upregulation of miR-143 by miRNA mimic suppressed the expression of extracellular signal-regulated kinase ERK5, mitogen-activated protein kinase MAP3K7, and cyclin D1, which further reduced breast cancer cell viability, while inhibition of miR-143 reversed these effects [63]. Wang et al. showed that miR-455 overexpression could inhibit breast cancer cell proliferation by a double-knockdown effect of Cdc2-related protein kinase CDK14 and cyclin D1 expression and also promoted expression of tumor suppressor p21 [64]. It was also shown that forced expression of commonly downregulated miR-424 in breast cancer cells enabled inhibition of cellular proliferation and regulation of the cell cycle by arresting cells in the G2–M cell phase. This study also showed that miR-424 gained its anti-oncogenic functions by binding to its selective target, cyclin-dependent kinase *CDK1*. In addition, the expression of the Yes-associated protein YAP, of the Hippo pathway, and p-ERK1/2 of the ERK pathway were also decreased upon miR-424 overexpression [65]. Furthermore, another study by Chen et al. provided evidence that miR-543 suppressed breast cancer cell proliferation, hindered cell cycling, and promoted cell apoptosis via direct regulating of ERK/MAPK pathway [66]. Huang et al. revealed that the overexpression of miR-26a-5p with miRNA mimic transfection induced proliferative growth of breast cancer cells, with a marked decreased in the expression levels of cell cycle regulators cyclin D1, CDK4, and CDK6, and increased the expression of p21, p27, and p53 tumor suppressor protein [67]. A subsequent study reported that the overexpression of miR-26a-5p also attenuated the ring finger protein RNF6/ERα/BCL-xL axis [67].

Yan et al. showed that upon the induction of estradiol, the expression level of MYC increased, resulting in transcriptional activation of a long non-coding RNA, PVT1, in breast cancer cells. Increased levels of PVT1 also significantly induced the expression of miR-1207-5p in breast cancer samples compared to non-cancerous controls. Increased miR-1207-5p expression promoted cell proliferation and increased the percentage of cells at G2 phase of cell cycle, whereas miR-1207-5p inhibition suppressed cell viability and cell cycle progression. Furthermore, overexpression of miR-1207-5p negatively regulated STAT2 expression, and further inactivated cell cycle-dependent kinase inhibitors CDKN1A and CDKN1B to promote cell cycle progression [68].

The markedly overexpressed miR-492 repressed the expression of transcription factor SOX7 in both transcriptional and translational levels, resulting in increased cell proliferation and cell cycle progression. As SOX7 is closely related to Wnt/β-catenin signaling activity, ectopic expression of miR-492 also led to upregulation of cyclin D1 and c-MYC. Additionally, Shen et al. showed that the percentage of cells at G0/G1 phase was decreased in miR-492-overexpressed breast cancer cells, whereas the percentage cells at S phase was increased, suggesting that miR-492 promoted the G1–S cell cycle transition [69].

Hua et al. stated that miR-135b was upregulated in breast cancer specimens and cell lines. Further overexpression of miR-135b with miRNA mimic resulted in increased cell proliferation and accumulation of S-phase and G2/M phase cells. This study also showed that miR-135b could promote cell growth and disrupt the cell cycle by negatively regulating LATS2 tumor suppressor kinase and the Hippo pathway in breast cancer cells. In addition, genes downstream of LATS2 and Hippo pathways, including cyclin-dependent kinase *CDK2* and *p-YAP*, were also regulated under the miR-135b/LATS2 axis [70].

Dysregulation of miRNAs in cell proliferative and cell cycle regulatory pathways is also attributed to resistance towards breast cancer treatments aimed at suppressing cell growth and proliferation [71]. Increased WBP2, which functions as the transcriptional coactivator of ERα/progesterone receptor (PR) transactivation, was associated with poor prognosis in ER+ breast cancer patients. Increased WBP2 expression also facilitated G1–S transition by regulating cell cycle-related proteins, including, p21, CDK4, and cyclin D1. Moreover, miR-206 overexpression and WBP2 knockdown reduced tamoxifen-resistance in breast cancer cells [72]. A study by Chu et al. also proved that suppressed expression of miR-15a/16 caused tamoxifen resistance in breast cancer cells by increasing the cell proliferation rate and cell cycle progression, while forced expression of miR-15a/16 re-sensitized breast cancer cells towards tamoxifen treatment by negatively inhibiting cyclin E1. In addition, increased expression of E2F7, which often correlated with higher relapse and poor prognosis in tamoxifen-treated breast cancer patients, was found to inhibit transcription of the miR-15a/16 cluster. In summary of these findings, overexpression of E2F7 resulted in decreased expression of the miR-15a/16 cluster, promoted cyclin E1 expression, and further induced cell growth via tamoxifen resistance [73]. Additionally, Liu et al. report the presence of a miR-26a/E2F7/MYC feedback loop in the regulation of tamoxifen resistance in ER+ breast cancer.

miR-26a was downregulated in ER+ breast cancer tissues whereas transcription factor E2F7 was upregulated. miR-26a overexpression by mimic transfection directly repressed E2F7 expression via translational inhibition, and indirectly inhibited MYC expression partly via E2F7 repression. E2F7 overexpression led to decreased expression of miR-26a through MYC-triggered transcriptional inhibition of miRNA. Resistance to tamoxifen was overcome with miR-26a overexpression and E2F7 silencing, which resulted in reduced breast cancer cell viability and G1 cell cycle arrest [74]. Studies by Tormo et al. suggested that overexpression of miR-26a and miR-30b was responsible for sensitizing HER+ breast cancer to trastuzumab treatment by inducing G1 arrest and decreasing the number of S and G2 proliferative cells. The ability of miR-26a and miR-30b to induce sensitization to trastuzumab treatment was shown to be via the effect of cyclin E2 silencing [75].

miR-365 [76] and miR-22 [77] also have decreased expression in breast cancer tissues relative to healthy, non-tumor tissues. Forced expression of both miR-365 and miR-22 through miRNA mimics gave rise to decreased breast cancer cell growth and increased sensitivity to fluorouracil and paclitaxel, respectively. miR-365 and miR-22 gain their functions in overcoming chemoresistance through respectively targeting GALNT4 and NRAS. GALNT4 is responsible for glycosylation-based post-transcriptional protein modification, whereas NRAS is an oncogenic activator of PI3K/Akt-, MAPK/ERK-, and NF-κB kinase-associated pathways; both are therefore important for cell proliferation and tumor progression [76,77].

Glucocorticoid treatment is frequently used as a pretreatment co-medication in chemotherapy [78]. A study by Senthil Kumar et al. found that treatment with either the synthetic glucocorticoid dexamethasone (DEX) or the natural glucocorticoid-mimicking compound, antcin A (ATA) markedly increased miR-708 expression in breast cancer cells via glucocorticoid receptor alpha (GRα) activation [79]. A decrease in the percentage of proliferative, viable breast cancer cells was observed following treatment with either DEX, ATA, or transiently transfected precursor miR-708 compared to the untreated controls, suggesting the inhibitory effects of DXE and ATA in breast tumorigenesis are via the miR-708/GRα axis. In addition, treatment with DXE and ATA also resulted in cycle arrest at the G2–M transition and G1–S transition phase, respectively. Further immunoblotting validation confirmed that DEX and ATA downregulated the G1–S transition regulatory proteins, notably cyclin D1, CDK4, and CDK6, whereas cyclin B and CDK1 were dramatically suppressed by ATA. However, both the DEX and ATA treatment did not affect the protein expression levels of cyclin A and cyclin E. Interestingly, upregulation of growth arrest regulatory proteins, p21^Cip1^ and p27^Kip1^, was also detected with either DEX or ATA treatment in breast cancer cells [79]. Furthermore, this study also showed that the activation of miR-708 by DXE and ATA was able to impair IKKβ expression but not that of IKKα nor IKKγ. The expression of the NF-κB-associated genes *COX-2* and *c-MYC* was also decreased upon miR-708 activation by GR agonist treatment or precursor miR-708 mimic transfection [79].

### 3.2. Metastasis and Invasion

Cancer cells have loose cell–matrix interactions in which they are less adhesive to the extracellular matrix compared than non-cancerous cells, allowing them to invade or metastasize via surrounding blood or lymphatic systems [80]. Epithelial–mesenchymal transition (EMT) is an important feature of the breast cancer metastasis cascade, enabling cancer cells to acquire stem-like features and facilitating their migratory and invasive capabilities [81]. EMT is known to involve loss of E-cadherin expression which further reduces cell localization or cell–cell contact [82]. There are also studies indicating that reduced levels of E-cadherin in cancer cells might potentiate the Wnt/β-catenin signaling pathway [83]. Overexpression of vimentin [84] and the expression switch of E-cadherin to N-cadherin is also a promoter of EMT that contributes to cancer cells’ metastatic phenotype [85].

There is a growing body of literature supporting the role of microRNAs in influencing the metastatic and invasive potential of breast cancer cells through the regulation of EMT and genes responsible for cell motility and invasion. Jin et al. discovered that overexpression of the miR-200c/141 cluster mediated the metastatic potential of breast cancer cells by positively upregulating the expression of SerpinB2. Overexpression of the miR-200c/141 cluster in breast cancer cells was also observed, with elevated mRNA expression of various transcription factor members, including *c-Jun*, *c-Fos*, and *FosB* mRNAs, nuclear import event of c-Jun, and induction of SerpinB2 promoter-directed chloramphenicol acetyltransferase (CAT) activity. Additionally, the expression of miR-124a and miR-26b, which directly targeted *SepinB2*, was downregulated in breast cancer cells. In a xenograft mouse model, miR-200c/141 overexpression promoted lung and lymph node metastasis, whereas siRNA-mediated SerpinB2 knockdown reverted the miR-200c/141-induced metastasis. SerpinB2 was highly associated with metastasis risk in breast cancer by overexpression in the triple negative breast cancer subtype (TNBC) compared to the luminal subtype. SerpinB2 was also correlated with increased metastatic potential and unfavorable outcomes in breast cancer patients. Thus, this study suggested that high expression of the miR-200c/141 cluster and SerpinB2 may serve as a prognostic indicator in TNBC cancer [86].

Other studies have found miRNAs that influence metastasis and cell invasion in breast cancer with their activity being induced by FOXP3 and KAT2B. Zhang et al. found that both members of the miR-200 family, miR-200c and miR-141, were induced by FOXP3, with KAT2B acting as the coordinator. In this study, the heterozygous *Foxp3 sf/+* breast cancer mouse model was used to analyze the regulation of mouse miR-200s during tumor progression. miR-200c/141 expression was low in tumor cells, but miR-200c/141was elevated in plasma during tumor progression and metastasis in these mice. In breast cancer patients, it was also confirmed that the plasma levels of miR-200c/141 were higher in metastatic breast cancers compared to localized breast tumors. Additionally, in these patients, the increased plasma miR-200c/141 appeared to originate from the tumor cells during cancer progression, suggesting a potential role for this molecule as a biomarker for breast tumor metastasis [87].

Shao et al. demonstrated the elevated expression of plasma miR-200a and miR-210 in chemotherapy resistance patients compared to chemosensitive patients. The expression of miR-200a was closely associated with breast cancer stage, with increased expression of miR-210 in advanced stage IV breast cancer. These authors also found that increased expression of miR-210 was correlated with liver, lung, and brain internal organ metastasis. The association between miR-200 and miR-210 and chemoresistance suggest the potential for these molecules as biomarkers of drug resistance [88]. Other studies have verified that the expression profiles of circulating miRNAs extracted from plasma were able to distinguish between breast cancer patients from localized luminal A group compared to patients with metastatic breast cancer. This study showed the overexpression of miR-331 in patients with metastatic breast cancer compared to patients with locally confined breast cancer or healthy controls. Contrastingly, underexpression of miR-195 was detected in breast cancer patients with metastases in comparison to patients with localized breast cancer group or healthy controls. Molecular studies confirmed the tumorigenic role of miR-331 is due to its association with gene targets related to metastatic processes including *HER2*, *HOTAIR*, *E2F1*, *DOHH*, and *PHLPP*. In addition, the role of miR-195, a known tumor suppressor, has also been validated by confirmation of its target genes, *FASN*, *HMGCR*, *ACACA*, and *CYP27B1*, which are implicated in tumor growth, EMT, invasion, and metastasis [89].

A study by Hong et al. also demonstrated the oncogenic properties of another member of the miR-200 family, miR-200b. miR-200b was shown to enhance breast cancer cells’ invasion and migratory ability via regulating Ezrin/Radixin/Moesin (ERM), a potential biomarker for breast tumor development. These data suggest that the underlying metastatic mechanisms moderated by overexpression of miR-200 members could be different for different miRNAs. While there is clear potential for miRNAs of the miR-200 family as biomarkers for breast cancer, further data are required to fully elucidate the mechanisms through which these molecules contribute to the metastatic process and how they could be advantageously used as biomarkers or treatment targets [90].

Rewiring of energy metabolism is widely regarded as a hallmark of breast cancer. It was reported that miR-122 is highly secreted by breast cancer cells into the circulation. The high levels of secreted miR-122 were attributed to reprogramming of glucose metabolism in the pre-metastatic niche. In this study, miR-122 was shown to suppress glucose uptake by non-tumor cells in order to accommodate the metabolic need of tumor cells. This process facilitated metastasis in vitro and in vivo, due to the increased availability of nutrients in the pre-metastatic niche. Fong et al. identified that the miR-122-induced decrease in glucose consumption in non-tumor cells was mediated by pyruvate kinase (PKM) and citrate synthase (CS) downregulation. In vivo systemic administration of anti-miR-122 improved glucose uptake by distant organs, including the brain and lungs, and decreased the rate of metastasis [91]. Wnt/β-catenin signaling has been demonstrated as one of the important regulators controlling the mechanism of EMT and cancer metastasis. Cai et al. reported that miR-374a expression was elevated in metastatic breast cancer cells and linked to a pro-metastatic phenotype in vitro. A spindle- or star-like cell morphology was observed in miR-374a-enriched breast cancer cell cultures. Additionally, aberrant expression of miR-374a was associated with substantial downregulation of epithelial markers, including E-cadherin, γ-catenin, and CK18, whereas the expression of mesenchymal markers, such as vimentin and N-cadherin, was significantly upregulated. These results suggested that miR-374a was associated with EMT features in breast cancer cells. Ectopic expression of miR-374a also enhanced distant metastasis in vivo. Studies indicated that miR-374a activated Wnt/β-catenin signaling cascades as its overexpression resulted in enhanced β-catenin nuclear translocation. In addition, miR-374a directly targeted and suppressed multiple negative regulators of the Wnt/β-catenin signaling pathway, including WIF1, PTEN, and WNT5A [92].

Contrastingly, Jiang et al. found that miR-148a was downregulated in breast cancer cells and tissues, and its overexpression by miRNA mimic decreased migration and invasion in breast cancer cells. WNT-1, which is one of the ligands in Wnt/β-catenin signaling, was identified as the target of miR-148a. In addition to the reduction of WNT-1 mRNA and protein levels, miR-148a overexpression also decreased expression of the other critical components of the Wnt/β-catenin pathway, including β-catenin, MMP-7, and TCF-4 in breast cancer cells. Additionally, miR-340 is another tumor suppressor miRNA that may inhibit the migration, invasion, and metastasis of breast cancer cells by targeting the Wnt/β-catenin signaling cascade [93]. Mohammadi-Yeganeh et al. identified miR-340 as functioning as a Wnt/β-catenin regulator miRNA by potentially targeting c-*MYC* and *CTNNB1* (encoding β-catenin) in Wnt/β-catenin-dependent and *ROCK1* in Wnt/β-catenin-independent signaling pathways (Rho/Rho-associated kinase (ROCK) signaling pathway). These studies show that specific miRNAs function as pivotal regulators in Wnt/β-catenin signaling pathway and therefore provide new insights into the molecular mechanisms of breast cancer metastasis [94].

The expression of miR-34a was downregulated in breast cancer specimens with lymph node metastasis and breast cancer cell lines, with further decreased expression in advanced clinical stages. Li et al. demonstrated that the expression level of miR-34a was inversely proportional to its direct target *TPD52*, a well-recognized oncogene in breast cancer. Knockdown of TPD52 by miRNA mimic in breast cancer cells increased E-cadherin expression levels while decreasing TGF-β and N-cadherin levels. In addition, repression of EMT and restrained breast cancer cell migration and invasion were observed following *TPD52* targeting by miR-34a. Furthermore, decreased miR-138 was associated with lymph node metastasis and invasion, whereas its overexpression led to inhibition of metastasis in breast cancer cells [95]. Zhang et al. carried out further studies on miR-138 in breast cancer cells, and found that miR-138 overexpression was involved in EMT inhibitory events via the impairment of vimentin, N-cadherin, and Snail expression, but with activation of E-cadherin expression [96].

Tissue from infiltrating ductal breast carcinoma displayed significantly weak expression of miR-494 relative to the strong positive miR-494 expression that was observed in normal, healthy breast tissue. Furthermore, elevated expression of miR-494 was strongly associated with increased expression of the epithelial marker, E-cadherin, which acts an important regulator of EMT and metastasis. Zhan et al. also showed that overexpression of miR-494 suppressed clonogenic and metastatic ability in vitro. Additionally, ectopic expression of miR-494 inhibited neoplasm initiation as well as pulmonary metastasis in vivo. Invasion of tumors into the peritoneal adipose tissue, abdominal muscle tissue, and lung metastasis was also extensively decreased in nude mice with miR-494 overexpression compared to the negative control. Further studies have determined that miR-494 gained its function by inhibiting *PAK1*, and restoration of *PAK1* expression was able to partially rescue miR-494 mediated inhibition of malignant propagation [97].

miR-33b was identified as a negative regulator of cell stemness and metastasis in breast cancer. In breast cancer cells, miR-33b was downregulated, and its expression negatively correlated with lymph node metastasis status in breast cancer patients. Ectopic overexpression of miR-33b in highly metastatic breast cancer cells inhibited cell stem-cell like properties, migration, and invasion in vitro, and suppressed lung metastasis in vivo. Conversely, miR-33b knockdown resulted in the opposite effects. Lin et al. showed that the mechanism of miR-33b-mediated inhibition of stemness or self-renewal, migration, and invasion of breast cancer cells was through the negative regulation of its downstream targets *HMGA2*, *SALL4*, and *Twist1* [98]. In addition, there are numerous other miRNAs that appear to be involved in the suppression of metastasis and invasion of breast cancer in vitro and in vivo; these include the miRNAs miR-497, miR-421, miR-193a, miR-211-5p, miR-335, miR-133a, and miR-124, which are proposed to suppress the expression of *SMAD7*, *MTA1*, *WT1*, *SETBP1*, *EphA4*, *LASP1*, and *STAT3*, respectively [99,100,101,102,103,104,105]. These miRNAs were downregulated in breast cancer tissues. Furthermore, re-introduction of the target genes reversed the inhibitory effects of these miRNAs on cell migration and invasion. In addition to its inhibition of SMAD7 expression, a recent study also showed that miR-497, together with miR-195, was able to directly target the 3′-UTR of cluster of differentiation *CD274* (or known as *PD-L1*) in TNBC cells, thus suggesting the potential of miR-497/195 in inhibiting the immune response and tumor immune escape [106,107]. Previous studies have also shown high accordance between CD274 expression and breast cancer metastases [107]. Additionally, Hong and team reported the downregulation of miR-204-5p in breast cancer cells. The overexpression of miR-204-5p resulted in a significant reduction in cell proliferation and migration in vitro, and inhibition of tumor growth and metastatic events in vivo. Subsequent studies on molecular mechanisms revealed that miR-204-5p is an important regulator of the PI3K/Akt signaling pathway by directly inhibiting *PIK3CB*, and its overexpression led to improved sensitivity towards PIK3CB inhibitors and chemotherapeutic drugs such as doxorubicin, taxanes, and bortezomib. Overexpression of miR-204-5p was also involved in tumor immune microenvironment remodeling or reprogramming by regulating key genes related to immune pathways, including TNF and cytokine signaling. In addition, a significant reduction was observed in the number of various immune cells in the tumor microenvironment, including myeloid-derived suppressor cells (MDSCs), macrophages, and natural killer (NK) cells as a result of miR-240-5p upregulation. By contrast, the overexpression of miR-240-5p resulted in an increase in the number of CD4^+^ T cells, CD8^+^ T cells, and regulatory T cells [108]. In summary, it is important that further research focuses on identifying the common metastasis and immune reprogramming regulatory pathways that mediate the crosstalk between these miRNAs and their candidate target genes in order to develop future prognostic and therapeutic strategies for anti-metastatic breast cancer treatment.

### 3.3. Apoptotic Response and Cell Death

Disruption to the chromosomal or genetic contents of a normal cell can lead to the induction of a signaling pathway of programmed cell death, known as apoptosis, and which serves as a defense mechanism [109]. Cancer cells are resistant to the apoptotic response even though their genetic contents are profoundly affected [110]. Disruption to any given point of the apoptosis pathway can trigger malignant transformation in mammary cells, enhancing cell viability [111]. It is shown that breast cancer cells are able to evade the apoptotic response through a number of mechanisms, including loss of tumor suppressor p53, dysregulation of caspase activity, upregulation of pro-survival regulators, downregulation of pro-apoptotic factors, and deactivation of death ligands [111]. Recent research has also shown that miRNAs play a significant role in the complex apoptotic regulatory mechanisms in breast cancer by targeting or initiating components involved in multiple cell death pathways [112].

Breunig et al. found that increased oncogenic miR-519a-3p expression in breast cancer cells enabled protection against the apoptosis-induced stimuli by TRAIL and Fas ligand, via diminishing the expression of its target genes coding for *TRAIL-R2 (TNFRSF10B)* and *caspase-8* and its indirect target gene for *caspase-7*. miR-519a-3p also compromised the anti-tumor functionality of natural killer (NK) cells by reducing granzyme B-induced apoptosis and negatively downregulating the expression of two key ligands for the NK cell-activating receptor NKG2D, MICA and ULBP2. The latter process enables cancer cells to avoid NK cell-mediated immune destruction by diminishing the recognition of MICA and ULBP2 on the cell surface by NK cells. Furthermore, miR-519a-3p was highly expressed in advanced-grade breast cancer with mutated p53 and associated with poor patient survival [113].

Sharma et al. found that the pro-apoptotic tumor suppressor p53 transcriptionally downregulated the expression of miR-191-5p through binding to the p53 response element present in its promoter region. In breast cancer cells, the overexpression of miR-191-5p resulted in a lower number of apoptotic bodies and a decrease in caspase-3/-7 activity, whereas anti-miR-191-5p reverted this effect. Moreover, the increased level of miR-191-5p was able to downregulate its potential target *SOX4*, which further reduced the expression of p53 in breast cancer cells, indicating the existence of a p53-miR-191-*SOX4* regulatory feedback loop. Additionally, it was found that anti-miR-191-5p treatment sensitized breast cancer cells towards apoptosis induced by the drug doxorubicin by increasing p53, suggesting the clinical potential of breast cancer therapeutics targeting this miRNA [114]. Wang et al. found that overexpression of miR-204 by miRNA mimic also promoted apoptosis in breast cancer cells by directly targeting *JAK2*. In addition, the expression level of miR-204 negatively correlated with p-STAT3 and its downstream anti-apoptotic proteins BCL-2 and survivin in breast cancer [115]. miR-148a is also associated with apoptosis, and is downregulated in breast cancer cells and tissues. Overexpression of miR-148a reduced the viability and chemoresistance of breast cancer cells and enhanced the apoptosis rate. Additionally, increased expression of miR-148a also prohibited tumor growth in vivo. The restoration of miR-148a was also found to suppress its direct target *BCL-2*. BCL-2 is responsible for preventing the release of mitochondrial apoptogenic factors into the cytoplasm, which further deactivates caspases, leading to induction of a pro-survival response. This suggests that miR-148a may serve as a potential tumor suppressor in breast cancer by silencing pro-survival BCL-2 [116].

Guan et al. report that miR-101 was downregulated in breast cancer cells and tissues, and transfection of miR-101 mimic resulted in a marked increase in apoptosis. A decrease in EYA1 expression was also observed following miR-101 mimic transfection, and transfection of miR-101 inhibitor gave the opposite result. In addition, expression of the components of Notch signaling pathways, including jagged1, Hes1, and Hey1 were significantly decreased following transfection with miR‑101 mimic and EYA1-siRNA. Together, these findings suggest that miR-101 promotes apoptosis by negatively targeting *EYA1* via Notch signaling pathways [117]. Other research suggests that miR-101 overexpression induces apoptosis in breast cancer in vitro and in vivo by targeting *SOX2*. In addition to the apoptotic response, suppression of SOX2 by miR-101 also resulted in inhibition of breast cancer growth, proliferation, and migration [118].

Another group of Chinese researchers discovered that kallistatin, an endogenous protein, was able to reduce viability and increase apoptotic cell death and caspase-3 activity in breast cancer cells. Kallistatin was also found to induce autophagy in breast cancer cells by increased expression of autophagy markers LC3B, Atg5, and beclin-1. Via its heparin-binding site, kallistatin can prompt autophagy by antagonizing Wnt3a-induced cancer cell proliferation and increasing PPARγ expression in breast cancer cells. Li et al. noted that kallistatin was able to inhibit the expression of oncogenic miR-21 by inhibiting the miR-21–Akt pathway, leading to reduced expression of anti-apoptotic BCL-2 and increased synthesis of pro-apoptotic BAX. Additionally, kallistatin reduced oncogenic miR-203 expression via PKC-ERK activation, with increased expression of tumor suppressor SOCS3. Conversely, the expression of the tumorigenic suppressors miR-34a and p53 was stimulated by kallistatin. These findings therefore suggest that kallistatin has contrasting effects in breast cancer cell death by suppressing the expression levels of miR-21 and miR-203, and stimulating miR-34a synthesis [119].

In mammals, the telomere consists of TTAGGG tandem repeats that are localized at the end of each chromosome and which serve to protect the chromosome against DNA damage and to prevent contact with neighboring chromosomes [120]. The length of telomeres progressively shortens during the course of cell division in human somatic cells, which finally leads to telomere dysfunction, chromosome instability, and the initiation of cellular senescence, apoptosis, and human aging [121]. Cancer cells have immortality due to their unique telomerase activity [121]. Telomerase is a ribonucleoprotein enzyme complex which maintains and replenishes the telomeric DNA repeats at the chromosomal end—one of the major tumor-promoting mechanisms in cancer [121]. The reverse transcriptase telomerase protein (hTERT) and the telomerase RNA template (hTERC) are essential regulators of telomerase activity [122]. A study by Dinami et al. showed that the expression of miR-296-5p and miR-512-5p, both of which target *hTERT*, was downregulated in breast cancer cells. Low miR-296-5p/512-5p expression and high hTERT expression are associated with poor clinical outcomes in basal-type breast cancer patients. Ectopic expression of miR-296-5p/512-5p was attributed to lower telomerase activity, weaker telomere maintenance, and the activation of replicative senescence and apoptosis programs in basal breast cancer cells, whereas epigenetic silencing of miR-296-5p/512-5p mediated the hTERT dependent proto-oncogenic effects of apoptosis protection in breast cancer cells [123]. The same group of researchers previously showed that miR-155 was also upregulated in breast cancer cells. miR-155 expression was shown to reduce the expression of its direct target *TRF1* at the telomeres [124]. TRF1 is one of the subunits of shelterin, also known as telosome, which is responsible for protection of the telomere [125]. Elevated expression of miR-155 also resulted in telomere fragility and genomic instability via inhibition of TRF1, and these are associated with poor clinical outcomes in ER^+^ breast cancer [124].

### 3.4. Hypoxia and Angiogenesis

As a tumor develops, it rapidly expands beyond the existing vasculature and leads to the formation of a tumor microenvironment of lower oxygen concentration compared to healthy tissues. This condition is known as hypoxia, which acts as a key regulator of angiogenesis in breast cancer [126]. It is thought that the sustained proliferation and growth of the breast tumor triggers neoangiogenesis, the formation of new blood vessels, in order to supply oxygen and nutrients to the tumor [127]. At the same time, the newly formed blood capillaries propagate the metastatic process by allowing easy penetration and infiltration of cancer cells [128]. HIF is a family of hypoxia-inducible transcription factors that regulates various critical breast cancer pathological processes, including stem cell homeostasis, cell proliferation, metastasis, and therapeutic resistance [129]. Additionally, VEGF is another important pro-angiogenic factor that stimulates the buildup of blood vessels by endothelial cells [130]. Hence, knowledge of the regulatory mechanisms of these hypoxia/angiogenesis-related genes by miRNAs could inform the development of promising anti-angiogenic agents for breast cancer.

miR-210 is the most consistently and significantly induced miRNA during hypoxia. Camps et al. performed a hypoxia timecourse miRNA sequencing data analysis on breast cancer cells. The upregulation of miR-210-3p was detected throughout the whole timecourse of the study. Additionally, upregulation of miR-210-3p was shown to be associated with HIF binding sites by HIF-1α and HIF-2α chromatin immunoprecipitation (ChIP)-sequence analysis [131]. Zhang et al. also reported that HIF-1α mRNA and miR-210 expression were markedly upregulated in a hypoxic environment [132]. Recent research by Costales et al. introduced a small molecule named Targapremir-210 that has binding affinity to the Dicer site of the miR-210 hairpin precursor. This interaction inhibited the processing of mature miR-210; reverted the repression of GPD1L, a hypoxia-associated protein negatively regulated by miR-210; reduced HIF-1α levels; and triggered the apoptotic response in TNBC cells under hypoxic conditions. Furthermore, Targapremir-210 also inhibited tumor growth in a hypoxic TNBC cancer mouse xenograft model [133]. Additionally, Harquail et al. observed elevated expression of miR-210 in breast cancer cells under hypoxic conditions. A concomitant decrease was also observed for Pax-5, a protein target of miR-210 which is an important regulator of EMT/MET transitioning [134].

Nagpal et al. reported that the direct target of HIF-inducible miR-191 under hypoxia is the mRNA coding for RNA binding protein, HuR. TGFβ-signaling pathways were stimulated as a consequence of miR-191 negatively regulating HuR expression. The levels of several TGFβ pathway genes, including *TGFβ2*, *SMAD3*, *BMP4*, *JUN*, *FOS*, *PTGS2*, *CTGF*, and *VEGFA*, were found to be higher in miR-191-overexpressing breast cancer cells. Finally, miR-191-inhibiting treatment led to drastic reduction in spheroid tumor volume [135].

Roscigno et al. noted an upregulation of miR-24 in breast cancer cells treated under hypoxic conditions. Overexpression of miR-24 in these cells led to increased formation of mammospheres, increased expression of *Nanog* and *Oct-3/4* stemness genes, and decreased expression of pro-apoptotic *BimL*. miR-24 was able to bind to its potential target *F1H1*, which encoded an asparaginyl β-hydroxylase that promotes transcriptional repression of HIFs. Therefore, F1H1 suppression, as mediated by miR-24, enabled HIF-1α protein stabilization and increased the levels of two HIF-1α direct targets, Snail and VEGFA. Contrastingly, overexpression of F1H1 reverted these miR-24-mediated effects [136].

Li et al. discovered that overexpression of miR-29b could hinder Human Umbilical Vein Endothelial Cells (HUVECs) formed by 3D capillary-like tubular structures, and tumor cell proliferation, migration, and formation. The systemic treatment of miR-29b suppressed tumor vascularization, inhibited infiltration of tumor-associated macrophages, inhibited tumor growth, and promoted the apoptotic response in vivo, without inducing cytotoxicity. It was also demonstrated that the role of miR-29b in anti-angiogenesis and anti-tumorigenesis was through functional targeting of Akt3 protein and inducing VEGF and c-MYC arrest in breast cancer cells [137].

Furthermore, Wu et al. demonstrated downregulation of miR-497 expression in breast cancer cell lines and clinical specimens. Overexpression of miR-497 by miR-497 mimic suppressed angiogenesis in vitro and in a nude mouse model by regulating VEGF and HIF-1α. miR-497 overexpression in vitro also disrupted the formation of capillary structures and led to a reduction in microvascular density (MVD) in vivo [138].

Lu et al. found that miR-140-5p was downregulated in advanced clinical stage and metastatic cancer tissues and associated with poorer prognosis. Overexpression of miR-140-5p by miRNA mimic resulted reduced the aggressiveness of breast cancers and also reduced angiogenesis in vitro and in vivo. The proposed mechanism for this effect was through targeted inhibition of VEGFA expression, together with decreased expression of other proteins, including CD31, Ki-67, and MMP-9 [139]. There is also evidence that miR-126 negatively regulates the pro-angiogenic protein VEGFA [140].

Recent research has demonstrated that mesenchymal stem cells (MSCs) can be recruited to the tumor microenvironment and promote tumor development through their interaction with tumor cells [141]. A growing body of evidence suggests that the regulation of tumor stroma by MSCs is via the secretion of extracellular vesicles such as exosomes [142]. MSC-derived exosomes have a key function in cell-to-cell communication by transferring their components, which include miRNAs [142]. Pakravan et al. found that the shuttling of miR-100, which is enriched in MSC-derived exosomes, was responsible for a significant downregulation in the expression and secretion of VEGF through modulation of the mTOR/HIF-1α signaling axis in breast cancer cells. Additionally, the inhibitory effects of MSC-derived exosomes on VEGF expression could be rescued with anti-miR-100 transfection, further supporting the role of exosomal shuttling of miR-100 in breast cancer. Furthermore, depletion of VEGF as mediated by MSC-derived exosomes was found to reduce the angiogenic behavior of endothelial cells in vitro by decreasing cell proliferation and migration and capillary tube formation. It therefore seems that exosomal miR-100 may serve as a potential angiogenic suppressor within the microenvironment of breast cancer cells [143].

**Figure 3:**
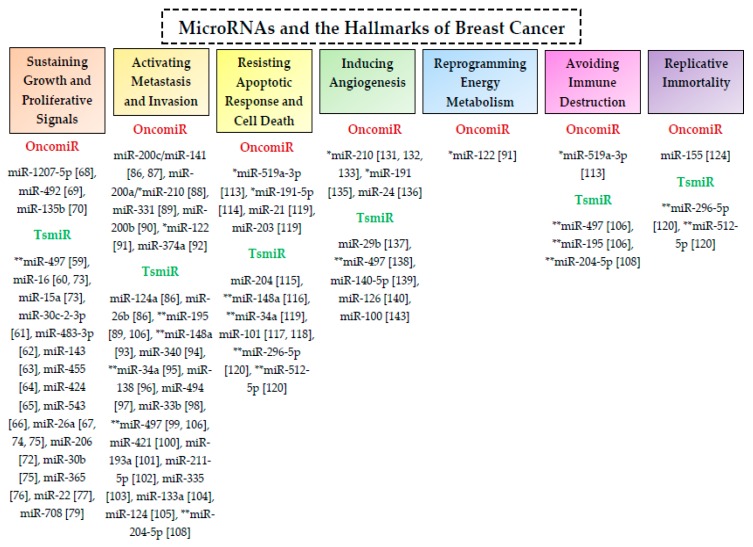
MicroRNAs involved in post-transcriptional regulatory interactions in breast cancer. The oncomiRs and tsmiRs associated with various hallmark characteristics of breast cancer are listed under the red and green subheadings, respectively. Each of these miRNAs can post-transcriptionally regulate a large number of genes involved in breast cancer. The symbols * and ** indicate the oncomiRs and tsmiRs, respectively, that regulate more than one of the hallmark features of breast cancer.

The oncomiRs and tsmiRs that involved in the post-transcriptional regulation of biological functions and hallmarks of breast cancer are summarized in Table 1 and Figure 3.

## 4. MicroRNAs and Breast Cancer Progression

Benign breast disease, such as atypical hyperplasia (AH), is associated with an increased risk of breast cancer [144]. It has been proposed that breast tumorigenesis is a multistep process which begins slowly with the development of clonally derived mammary cells, leading to AH, which then further develops into carcinoma in situ (CIS), and finally into invasive carcinoma [145]. Early detection of cancer at the stage of AH hyperplasia is therefore timelier for surgical intervention and is likely to improve patient survival rate. However, at present, there are no biomarkers that can accurately diagnose AH [146].

An et al. examined whether serum miRNAs could serve as biomarkers for discriminating between patients with AH or early-stage breast cancer patients compared with healthy patients or patients with benign proliferative tumors. From the pool of analyzed miRNAs, only miR-24 and miR-103a showed significant downregulation in AH and early-stage breast cancer. By contrast, a slight increase was observed for these miRNAs in the serum of benign proliferative tumor patients compared with healthy individuals. However, there was no significant relationship between either of these miRNAs and TNM Classification of Malignant Tumors (TNM) staging or clinical molecular subtypes, even though their regulation was slowly reduced with the progression of breast cancer (ductal carcinoma in situ (DCIS), I, II). In addition, analyses employing gene ontology (GO) enrichment analysis and Kyoto Encyclopedia of Genes and Genomes (KEGG) pathways revealed that the candidate gene targets of miR-24 and miR-103a were potentially involved in critical molecular signaling pathways of breast tumorigenesis, such as gene expression regulation, apoptotic processes, and Wnt and Notch signaling pathways. Interestingly, the key proteins of miRNA biogenesis, such as Dicer 1, Ago1, and Ago4, are also predicted target genes for miR-103a, suggesting a stem-loop feedback mechanism of regulation. However, this research was limited by a small sample size and the lack of a detailed exploration of the mechanisms behind this miRNA’s involvement in breast cancer progression [147].

Additionally, Stankevicins et al. performed a miRNA microarray-based global expression analysis on a series of 21T cell line representing different stages of breast cancer. This analysis showed that only miR-205-5p was markedly downregulated in the metastatic, invasive cell lines (21MT-1 and 21MT-2) relative to the localized, non-proliferative cells (21PT and 21NT). The reduced expression of miR-205-5p was also associated with advanced histopathological tumor grade and increased invasion rates in a Boyden chamber cell migratory assay. Although not statically significant, transfection of metastatic cells (21MT1 and 21MT2 cells) with miR-205-5p precursor resulted in reduced migratory potential, whereas healthy and in situ cells (H16N2, 21PT and 21NT cells) transfected with miR-205-5p inhibitor showed partial elevation in migration rate. miR-205-5p was also predicted to target genes responsible for breast cancer invasiveness, including *SOCS3*, *PTPRN2*, and *MMP3*, and also genes associated with EMT regulatory functions, such as *TGFB1*. Therefore, this work showed that miR-205-3p may serve as a key player during progression of a tumor to an invasive, metastatic phenotype [148].

In summary of the above findings, dysregulation of miRNA expression appears to play a pivotal role in the transition from non-proliferative cellular conditions to a cancerous state. Most of the published research conducted into miRNAs in the context of breast cancer only considers established, late-stage breast cancer. Given the potential of miRNAs as sensitive biomarkers for early-stage cancer such as benign breast disease, in which disease prognosis is considerably more favorable, more research is needed to investigate the regulatory impact of these molecules specifically in early-stage breast cancer.

## 5. MicroRNAs Act in Networks in Their Regulation of Breast Cancer

As discussed above, a single miRNA or miRNAs can critically regulate genes controlling various pathobiological processes implicated in cancer. However, most of the studies discussed above focused on the role of miRNA(s) solely as discrete entities, without considering the complex, interconnected nature of the breast cancer ‘miRNome’. As any given miRNA is predicted to target up to thousands of mRNA transcripts, it is a profound challenge to identify the miRNA or group of miRNAs that are the most prominent regulators of breast cancer pathological processes. In fact, to date, there are no clear miRNA expression profiles that are interlinked between various human cancers. As an example, miR-498 can serve as a potential oncomiR in both breast cancer and prostate cancer by targeting the tumor suppressor gene *PTEN* [149]. At the same time, miR-498 functions as a tsmiR in liver cancer by targeting the oncogene *ZEB2* [150]. In breast cancer cells, other oncomiRs, miR-1297 and miR-103b, are also responsible for breast tumorigenesis by directly targeting *PTEN* [151,152], whereas miR-221 and miR-19a-3p also displayed potent oncogenic roles through negative regulation of *PTEN* in lung and hepatocellular carcinoma, respectively [153,154]. Therefore, it is worthwhile to investigate or refine the molecular co-relation in terms of miRNAs, their target genes, and the respective signaling pathways in future studies for better prognostication, detection, and treatment of human cancers. In addition, attention should also be given to discoveries that focus on the unique and novel miRNA regulatory trends in breast pathobiology.

A study by De Anda-Jáuregui et al. identified a set of five miRNAs, miR-190b, miR-let-7i, miR-292-b, miR-511, and miR-141 (also known as Commodore miRs; Cdre-miRs), that were non-redundant and highly interconnected in regulating a large network of associated genes in breast cancer [155]. It was found that miR-190b regulated a group of genes involved in dynein assembly, vitamin metabolism, and mammary gland epithelial cell proliferation. Genes associated with melanocyte transport, angiogenesis, and epithelial cell migration were regulated by miR-292-b; genes of motility, migration, and extracellular matrix organization were controlled by miR-141; genes of cytokine production and cell activation were under the regulation of miR-511; whereas gene neighbors related to adaptive immune response and leukocyte cell–cell adhesion were under modulation by miR-let-7i. There was a lack of overlaps between the gene neighborhoods regulated by each Cdre-miR, but both miR-511 and miR-let-7i Cdre-miRs were responsible for co-regulation of the innate immunity process. As each Cdre-miR is an important control element of specific biological processes, their removal or alteration will lead to the disconnection of a group of genes which then impair certain biological functionality [155].

The action of these non-redundant Cdre-miRs in the form of highly interconnected networks is a property that is only found in the context of breast cancer. Interestingly, no Cdre-miR transcripts have been identified in healthy breast tissue. Therefore, Cdre-miRs could potentially serve as novel biomarkers in monitoring the transcriptional regulatory perturbations observed in breast cancer, and even as attractive therapeutic targets [155].

## 6. Therapeutic Potential and Delivery Options of MicroRNAs in Breast Cancer

Owing to their small molecular size and ability to regulate the expression of genes associated with the progression of various cancers, miRNAs have the potential to revolutionize breast cancer therapeutics [156]. Potential miRNA-based breast cancer therapies are generally based on the approaches of either silencing oncogenic miRNAs via miRNA inhibitors or restoring the functions of tumor suppressor miRNAs via miRNA mimetics. Experimental studies of the potential therapeutic utility of miRNA-focused approaches have, to date, generally employed in vitro techniques to quantify their effects on mRNA and protein concentrations.

miRNA inhibition therapy can be used to restore the normal expression and function of the target tumor suppressor genes by inhibition of the normally upregulated oncogenic miRNAs in human breast cancers. miRNA antagonists or antagomirs are complementary single-stranded and chemically modified oligonucleotides that can expropriate or competitively inhibit the endogenous oncogenic miRNAs from being recognized and processed by RISC. This renders the inhibited miRNAs no longer able to recognize or interact with their target tumor suppressor mRNAs [157]. Antagomirs such as 2′-*O*-methyl modified oligonucleotides, locked nucleic acid (LNA) anti-miRs, and cholesterol-conjugated antagomirs are among the miRNA inhibitors that are widely used in miRNA inhibition therapy [158]. In addition, there has also been interest in the therapeutic potential of miRNA sponges which contain multiple artificial miRNA binding sites that can competitively bind and inhibit specific miRNAs or clusters of miRNAs [159]. By contrast, miRNA masks can bind to the target mRNA and selectively inhibit the interaction with the specific miRNA [160]. These masking nucleic acids can precisely mask the mRNA from the endogenous miRNA and thus prevent its suppression.

In miRNA replacement therapy, the normal function of the tumor-suppressive miRNAs can be re-established by replacing or substituting the downregulated miRNAs by employing miRNA-like synthetic molecules known as miRNA mimics [157]. These miRNA mimics are small, chemically modified 2′-*O*’-methoxy RNA duplexes that can be loaded into RISC, mimicking the function of endogenous miRNAs by inhibiting the target mRNAs that are commonly oncogenic [157].

In general, miRNA modulators have low stability; the naked RNAs tend to be degraded by nucleases and removed from the body by renal excretion [161]. Thus, the specific, efficient, and safe delivery of miRNA modulators to the tumor sites is crucial for the success of miRNA-based cancer therapeutic strategies. Effective miRNA delivery has been shown by employing various viral vectors, such as lentivirus, retrovirus, adenovirus, and adeno-associated virus (AAV), expressing miRNA antagonists or mimics [162]. In addition, nanostructured lipid carriers, such as liposomes consisting of lipid bilayers, have also been successfully employed; these encapsulate the miRNA antagonists or mimics, protecting them from nuclease degradation and increasing the stability of their delivery into the cells [163]. Nanoparticles such as altered polyethylene glycol (PEG), inorganic nanoparticles (iron, gold, carbon, silica), and nanoparticles with targeting molecules such as ligands, peptides, or antibodies can also be used for effective miRNA delivery [156]. Furthermore, polymer-based methods also have potential due to their biodegradability and high electrostatic affinity for cellular membranes [164]. miRNA delivery has also been achieved using synthetic polyethylenimine (PEI), poly (lactic-co-glycolic acid) (PLGA), and other natural cationic polymers such as chitosan and atelocollagen [165].

CRISPR (clustered regularly interspaced short palindromic repeats)-Cas 9 (CRISPR-associated protein 9) is a newly emergent genome editing approach that has gained widespread attention as a technique for full and permanent gene knockout [166]. CRISPR-Cas 9 has been used for editing of protein-coding genes in breast cancer, including *HER2* [167] and *MIEN1* [168]. However, editing of non-protein coding RNA genes (including miRNAs) using this approach has received relatively little attention. Most of the currently published strategies of miRNA replacement have relied on transient transfection of miRNA inhibitors or antagomiRs into the cells [169], only promoting their expression for a finite period of time without integration into the cell genome [170]. Additionally, it has been suggested that the transfected material is likely to be degraded by nucleases or diluted following cell division [170]. However, in a recent study by Hannafon et al., CRISPR-Cas9 deletion of miR-23b and miR-27b was performed in order to study their regulation of breast cancer cells [171]. The genomic knockdown of miR-23b/27b resulted in reduced cell proliferation rate, formation of fewer cell colonies, and the attenuation of anchorage-independent growth in vitro. Furthermore, the growth rate and tumor volume of xenografts from miR-23b/27b knockout mice was dramatically reduced compared to control mice treated with empty vectors. These data therefore suggest that editing of genes encoding endogenous miRNAs is a potentially revolutionary therapeutic approach for breast cancer.

## 7. Conclusions

There is a vast and growing literature that firmly supports the involvement of miRNAs in cancers such as breast cancer. It is becoming more widely acknowledged that these molecules play important roles in regulating gene expression in order to achieve homeostasis, and that dysregulation of their activity can lead to adverse consequences in a wide range of disease pathways. There is therefore a great potential for miRNA-based therapeutics to serve as highly specific approaches or targeted therapies for breast cancer treatment. This potential is evidenced in in vitro studies, which show that miRNA-based techniques can modulate the expression of target genes in a highly specific and effective way. However, there are a number of challenges to overcome in order to successfully translate these promising laboratory results into efficacious therapies in clinical practice. These factors include the development of more efficient delivery options, the issue of degradation or instability, potential off-target effects, and the long-term safety of these agents in vivo. Furthermore, the underlying mechanisms that govern the interactional networks between miRNAs and the human genome, transcriptome and proteome have to be clearly understood before their effective transition into medical or pharmaceutical settings. It is therefore important that the wider consequences of candidate miRNA-based therapies are studied in vivo within a complex biological system, using multiomics approaches, for example. Additionally, among the dysregulated miRNAs in breast cancer specifically, it is important to determine the most representative miRNA or groups of mRNAs at each stage of the disease; this will help to identify and prioritize the most promising treatment targets, with emphasis on developing strategies to detect and treat breast cancer earlier. In conclusion, the advancement in miRNA-based therapeutics has the potential to revolutionize and personalize breast cancer treatment. A more in-depth knowledge of the mechanisms of action and the wider biological consequences of miRNA-based therapies is first required, but these should not be considered insurmountable barriers to combatting this common and devastating disease.

## Figures and Tables

**Table 1:** MicroRNAs involved in the regulation of breast cancer.

MicroRNA	Interacted/Correlated Gene(s) and Protein(s)	Associated Events	Reference
Major oncogenic microRNAs in breast cancer			
miR-1207-5p	STAT2, CDKN1A, CDKN1B	Promotion of cell proliferation and G2 cell cycle progression	[68]
miR-492	SOX7, cyclin D1, c-MYC	Promotion of cell proliferation and G1–S cell cycle progression	[69]
miR-135b	LATS2, CDK2, p-YAP	Promotion of cell proliferation and S–G2/M cell cycle progression	[70]
miR-200c and miR-141	SerpinB2, c-Jun, c-Fos, FosB, FOXP3, KAT2B	Promote metastasis and elevated in serum of metastatic mouse model and breast cancer patients	[86,87]
miR-331	HER2, HOTAIR, E2F1, DOHH, PHLPP	Promotion of metastasis and invasion by elevation in plasma of metastatic breast cancer patients	[89]
miR-200b	Ezrin/Radixin/Moesin (ERM)	Promotion of metastasis and invasion	[90]
miR-122	pyruvate kinase (PK) and citrate synthase (CS)	Promotion of metastasis by reprogrammed glucose metabolism	[91]
miR-374a	E-cadherin, γ-catenin, CK18, vimentin, N-cadherin, Β-catenin, WIF1, PTEN, WNT5A	Promotion of metastasis by regulating EMT and Wnt/β-catenin signaling	[92]
miR-519a-3p	TRAIL-R2 (TNFRSF10B), caspase-8, caspase-7, MICA, ULBP2	Promotion of apoptosis resistance and escape from natural killer cell recognition	[113]
miR-191-5p	SOX4, caspase-3, caspase-7, p53	Promotion of apoptosis resistance and doxorubicin resistance	[114]
miR-21	Akt, BCL-2, BAX	Pro-survival effect can be overcome by kallistatin	[119]
miR-203	PKC-ERK, SOCS3	Pro-survival effect can be overcome by kallistatin	[119]
miR-155	TRF1	Telomere fragility and genomic instability	[124]
miR-210	HIFs, GPD1L, Pax-5	Hypoxia-inducible miRNA	[131,132,133]
miR-191	HuR, TGFβ2, SMAD3, BMP4, JUN, FOS, PTGS2, CTGF, VEGFA	Hypoxia-inducible miRNA and stimulator of TGFβ-signaling pathways	[135]
miR-24	Nanog, Oct-3/4, BimL, F1H1, HIF-1α, Snail, VEGFA	Hypoxia-inducible miRNA	[136]
Major tumor suppressor miRNAs in breast cancer			
miR-497	Cyclin E1	Anti-proliferative and G1-S cell cycle arrest	[59]
	SMAD7	Anti-metastasis and anti-invasion	[99]
	CD274	Anti-metastasis, anti-tumorigenic and inhibition of immune response or tumor immune escape	[106]
	VEGF, HIF-1α	Anti-angiogenesis and anti-tumorigenic	[138]
miR-16	Cyclin E1, E2F7	Anti-proliferative and G1–S cell cycle arrest, restores tamoxifen sensitivity	[60,73]
miR-30c-2-3p	Cyclin E1	Anti-proliferative and G1–S cell cycle arrest	[61]
miR-483-3p	Cyclin E1, p-NPAT, CDK2	Anti-proliferative and G1–S cell cycle arrest	[62]
miR-143	ERK5, MAP3K7, Cyclin D1	Anti-proliferative	[63]
miR-455	CDK14, Cyclin D1, p21	Anti-proliferative	[64]
miR-424	CDK1, YAP, p-ERK1/2	Anti-proliferative and G2–M cell cycle arrest	[65]
miR-543	ERK/MAPK	Anti-proliferative, cell cycle arrest and apoptosis	[66]
miR-26a	Cyclin D1, CDK4, CDK6, p21, p27, p53, RNF6/ERα/BCL-xL, E2F7, MYC, cyclin E2	Anti-proliferative, G1 cell cycle arrest and restores sensitivity to tamoxifen and trastuzumab treatment	[67,74,75]
miR-206	WBP2, p21, CDK4, cyclin D1	Anti-proliferative, cell cycle arrest and restores sensitivity to tamoxifen treatment	[72]
miR-15a	Cyclin E1, E2F7	Anti-proliferative and G1–S cell cycle arrest, restores tamoxifen sensitivity	[73]
miR-30b	Cyclin E2	Anti-proliferative, G1 cell cycle arrest and restores sensitivity to trastuzumab treatment	[75]
miR-365	GALNT4	Anti-proliferative and restores sensitivity to Fluorouracil chemotherapeutic treatment	[76]
miR-22	KRAS	Anti-proliferative and restores sensitivity to Paclitaxel chemotherapeutic treatment	[77]
miR-708	IKKβ, COX-2, c-MYC	Anti-proliferative and regulates cell cycle arrest upon induction of glucocorticoid agonists, DEX and ATA	[79]
miR-124a and miR-26b	SerpinB2	Anti-metastasis and anti-invasion	[86]
miR-195	FASN, HMGCR, ACACA, CYP27B1	Anti-metastasis and anti-invasion by underregulation in plasma of metastatic breast cancer patients	[89]
	CD274	Anti-metastasis, anti-tumorigenic and inhibits immune response or tumor immune escape	[106]
miR-148a	WNT-1, β-catenin, MMP-7, TCF-4	Anti-metastasis and anti-invasion by regulating Wnt/β-catenin signaling pathway	[93]
	BCL-2, caspases	Promotes apoptotic response and overcomes chemoresistance	[116]
miR-340	c-MYC, CTNNB1, ROCK1	Anti-metastasis and anti-invasion by regulating Wnt/β-catenin and Rho/Rho-associated kinase (ROCK) signaling pathways	[94]
miR-34a	TPD52, E-cadherin, TGF-β, N-cadherin	Anti-metastasis and anti-invasion by regulating EMT	[95]
	P53	Pro-apoptotic effect can be induced by kallistatin	[119]
miR-138	E-cadherin, vimentin, N-cadherin, Snail	Anti-metastasis and anti-invasion by regulating EMT	[96]
miR-494	PAK1, E-cadherin	Anti-metastasis and anti-invasion	[97]
miR-33b	HMGA2, SALL4, Twist 1	Anti-metastasis and anti-invasion	[98]
miR-421	MTA1	Anti-metastasis and anti-invasion	[100]
miR-193a	WT1	Anti-metastasis and anti-invasion	[101]
miR-211-5p	SETBP1	Anti-metastasis and anti-invasion	[102]
miR-335	EphA4	Anti-metastasis and anti-invasion	[103]
miR-133a	LASP1	Anti-metastasis and anti-invasion	[104]
miR-124	STAT3	Anti-metastasis and anti-invasion	[105]
miR-204-5p	PIK3CB	Anti-metastasis, anti-tumorigenic, restores sensitivity towards PIK3CB inhibitors and chemotherapeutic drugs (i.e., doxorubicin, taxanes and bortezomib), and involved in tumor immune microenvironment remodeling	[108]
miR-204	JAK2, BCL-2, survivin	Promotion of apoptotic response	[115]
miR-101	EYA1, jagged1, Hes1, Hey1, SOX2	Promotion of apoptotic response by negatively regulating Notch pathway	[117,118]
miR-296-5p and miR-512-5p	hTERT	Reduction of telomerase activity, impairment of telomere maintenance and activation of replicative senescence and apoptosis programs	[120]
miR-29b	Akt3, VEGF, c-MYC	Anti-angiogenesis and anti-tumorigenesis	[137]
miR-140-5p	VEGFA, CD31, Ki-67, MMP-9	Anti-angiogenesis and anti-tumorigenesis	[139]
miR-126	VEGFA	Anti-angiogenesis and anti-tumorigenesis	[140]
miR-100	VEGF, mTOR/HIF-1α	Shuttling of miRNA enriched in MSC-derived exosomes, anti-angiogenesis and anti-tumorigenesis	[143]

## Abbreviations

miRNA	microRNA
mRNA	messenger RNA
oncomiR	oncogenic miRNA
tsmiR	tumor suppressor miRNAs
